# Effects of IL-11/IL-11 Receptor Alpha on Proliferation and Steroidogenesis in Ovarian Granulosa Cells of Dairy Cows

**DOI:** 10.3390/cells12040673

**Published:** 2023-02-20

**Authors:** Hanxiao Wu, Peihao Sun, Ce Lv, Xinzhe Zhao, Mingxiao Liu, Qunli Zhou, Jiaomei Tang, Liguo Yang, Aixin Liang

**Affiliations:** 1 Key Laboratory of Agricultural Animal Genetics, Breeding and Reproduction of Ministry of Education, College of Animal Science and Technology, Huazhong Agricultural University, Wuhan 430070, China; 2 College of Veterinary Medicine, Northwest A&F University, Xianyang 712100, China; 3 National Center for International Research on Animal Genetics, Breeding and Reproduction, Huazhong Agricultural University, Wuhan 430070, China

**Keywords:** granulosa cell, interleukin-11, interleukin-11 receptor, proliferation, steroidogenesis

## Abstract

Granulosa cells (GCs) are essential for follicular growth, oocyte maturation, and steroidogenesis in the ovaries. Interleukin (IL)-11 is known to play a crucial role in the decidualization of the uterus, however, the expression of the IL-11 system (IL-11, IL-11Rα, and gp130) in the bovine ovary and its exact role in GCs have not been extensively studied. In this study, we identified the IL-11 signaling receptor complex in the bovine ovary and investigated the regulatory effects and underlying mechanism of IL-11Rα on the proliferation and steroidogenesis of GCs. We observed that the IL-11 complex was highly expressed in the GCs of large follicles. IL-11Rα knockdown significantly inhibited GC proliferation by inducing cell cycle arrest at the G1 phase, along with a significant downregulation of proliferating cell nuclear antigen (PCNA) and Cyclin D1 (CCND1) protein, and induced GC apoptosis by significantly upregulating the ratio of BCL-2-associated X protein (*BAX*) and B-cell lymphoma-2 (*BCL-2*). In addition, IL-11Rα knockdown attenuated the Janus kinase (JAK) 1–signal transducer and activator of transcription 3 (STAT3) signaling, which is related to cell proliferation and apoptosis. Furthermore, the enzyme-linked immunosorbent assay (ELISA) indicated that IL-11Rα silencing decreased the basal and forskolin (FSK)-stimulated secretions of estradiol and progesterone in GC culture medium concomitantly with a remarkable decrease in cytochrome P450 family 19 subfamily A member 1 (CYP19A1) and steroidogenic acute regulatory protein (StAR). We subsequently determined that this reduction in steroidogenesis was in parallel with the decrease in phosphorylations of protein kinase A (PKA) substrates, cAMP-response element binding protein (CREB), extracellular regulated protein kinase (ERK) 1/2, and p38 mitogen-activated protein kinase (MAPK). Taken together, these data indicate that the effects of IL-11/IL-11Rα on the proliferation and steroidogenesis in bovine GCs is mediated by the JAK1-STAT3, PKA-CREB, p38MAPK, and ERK1/2 signaling pathways. Our findings provide important insights into the local action of the IL-11 system in regulating ovarian function.

## 1. Introduction

In mammals, ovarian follicular development is a complex physiological process and is regulated by several factors, including hormones, growth factors, cytokines, and chemokines. Among the cytokines, interleukin (IL)-6 has been often thought to modulate ovarian function. For example, the concentration of IL-6 is not only associated with follicular growth and oocyte maturation but also with angiogenesis and the formation of the corpus luteum [1,2,3,4,5]. IL-11, another member of the IL-6 family, is a pleiotropic cytokine produced by fibroblast, endothelial, and epithelial cells [6,7]. Studies have demonstrated that IL-11 is involved in hematopoiesis, macrophage proliferation and differentiation, and mucosal protection. In the reproductive tract, IL-11 is important for the decidualization of the uterus. One study found that female mice were infertile due to the decidualization defect caused by the null mutation of the IL-11 receptor alpha chain (IL-11Rα) [8,9,10,11]. The actions of IL-11 are mediated through a receptor complex composed of the IL-11Rα and β-subunit receptor, glycoprotein 130 (gpl30) [12]. Although IL-11 shares gp130 as a signaling transductor with IL-6, it can also exert unique biological action through its individual receptor, IL-11Rα [13,14]. In classical signaling, the binding of IL-11 to the IL-11α receptor triggers the homodimerization of the gp130 subunit and the formation of a hexameric complex, leading to the activation of Janus kinase (JAK) [12,15]. Phosphorylated JAKs activate the signal transducer and activator of transcription 3 (STAT3), which then translocates to the nucleus, where it acts as a transcriptional activator of various target genes associated with cell proliferation and survival [13,16,17,18]. Apart from the standard JAK/STAT3 signaling pathway, the IL-11 complex is also related to other pathways, including the mitogen-activated protein kinase (MAPK) pathway [19], MEK/ERK pathway [18], phosphatidylinositol-3 kinase (PI3K)/AKT pathway [20], and Src-family kinase signaling pathway [21].

In the ovaries, IL-11 is secreted from both theca and granulosa cells, and a higher concentration of IL-11 has been detected in human follicular fluid from preovulatory follicles compared to other follicles [22]. In a recent study, the treatment of preovulatory follicles cultured in vitro with IL-11 stimulated progesterone production and steroidogenic acute regulatory protein (StAR) gene expression in rats [23]. Moreover, clinical studies have demonstrated that concentrations of IL-11 in follicular fluid increase in patients with polycystic ovary syndrome (PCOS) [24] and ovarian hyperstimulation syndrome (OHSS) [25]. Although the above studies suggest that IL-11 is a potential regulator in the physiological and pathological processes of the ovaries, the exact effect of the IL-11 system on bovine GCs has not yet been studied.

In the present study, we determine the expression of the IL-11 complex (IL-11, IL-11Rα, and gp130) in the bovine ovary and GCs. We also investigate the effects of the IL-11 complex on proliferation and steroidogenesis in the GCs of large follicles. Furthermore, the underlying molecular mechanisms mediated by the IL-11 system in GCs are determined. Briefly, IL-11/IL-11Rα regulate the proliferation and steroid production of bovine GCs through the JAK1-STAT3, PKA-CREB, p38MAPK, and ERK1/2 signaling pathways. These findings will help to increase our knowledge of the functional role of the IL-6 family in modulating folliculogenesis.

## 2. Material and Methods

### 2.1. Ethics Statement

All animal experiments in this study were approved by the Scientific Ethics Committee of Huazhong Agricultural University (HZAUCA-2021-0004) and were performed in accordance with the Guidelines for the Care and Use of Laboratory Animals of the Research Ethics Committee, Huazhong Agricultural University.

### 2.2. Tissue Collection

For analysis of tissue expression patterns, different bovine tissues from various organs, including the heart, liver, spleen, lung, kidney, brain, stomach, intestine, uterus, ovary, and muscle, were collected from healthy cows (n = 3) at a local slaughterhouse (Wuhan, Hubei, China). For cell cultures, ovaries were also harvested from cows at the same slaughterhouse (Wuhan, Hubei, China). All tissues were shipped to the laboratory within 2 h in phosphate-buffered saline (PBS) containing 1% penicillin–streptomycin solution (Biosharp, Hefei, China, Cat#BL505A) in a 37 °C incubator.

### 2.3. Cell Culture

After the ovaries were sterilized with 75% alcohol, GCs and follicular fluid (FF) from large (≥10 mm), medium and small follicles (2–9.9 mm) were aspirated using 25-gauge needles. After centrifugation at 1000× *g* for 10 min, the GCs and follicular fluid were separated. The GCs were washed twice in DMEM/high-glucose medium containing 1% penicillin–streptomycin solution, seeded at 1 × 10^5^ densities in medium plates with culture medium containing 10% fetal bovine serum (FBS, Gibco, Carlsbad, CA, USA, Cat#10270-106) and 1% penicillin–streptomycin solution, and cultured in a humidified incubator (Thermo Fisher Scientific, Waltham, MA, USA) at 37 °C with 5% CO_2_.

After transfection with IL-11Rα siRNA and negative control siRNA for 6 h or supplementation with 10 ng/mL of IL-11 (PeproTech, Cranbury, NJ, USA, Cat#200-11), the culture medium was replaced with serum-free medium supplemented with 1% penicillin–streptomycin solution, 1% insulin–transferrin–selenium (ITS) Liquid Media Supplement (Meilunbio, Dalian, China, Cat#PWL083), 0.1% bovine serum albumin (BSA) solution (Solarbio, Beijing, China, Cat#H1130). In addition, 200 nM androstenedione (Sigma-Aldrich, St. Louis, MO, USA, Cat#A-075) and 10 μM forskolin (Sigma-Aldrich, St. Louis, MO, USA, Cat#F6886) were added to the culture medium for steroidogenesis experiments. 

### 2.4. Interference Fragment Synthesis and Transfection

According to the sequence of the bovine IL-11Rα gene (NM_001034339.1), the IL-11Rα siRNA and negative control RNA oligonucleotides (siRNA-NC) were designed and synthesized by GenePharma Co., Ltd. (Shanghai, China) with the following sequence: siIL-11Rα: 5′-GCACUGACGAGGGCACCUATT-3′; siRNA-NC 5′-UUCUCCGAACGUGUCACGUTT-3′. All siRNAs were used at a final concentration of 30 nM.

GCs were cultured to 80 % confluence and then co-transfected with a mixture of 4 μL of jetPRIME (Polyplus Transferion^®^SA, Illkirch, France, Cat#114-15) and 3 μL of IL-11Rα siRNA or siRNA-NC for each well according to the transfection reagent kit’s instructions. 

### 2.5. Cell Viability Assay

The viability of GCs was detected using Cell Counting Kit-8 (CCK-8, Dojindo, Kumamoto, Japan, Cat#CK04) according to the manufacturer’s instructions. Briefly, the cells were seeded in 96-well plates. After transfection for 24 and 48 h, 90 μL of FBS-free medium with 10 μL of CCK-8 solution was added to each well and the samples were incubated for 2 h. Finally, the absorbance at 450 nm was measured using a microplate reader (PerkinElmer, Waltham, MA, USA).

### 2.6. Cell Apoptosis Analysis

Apoptosis of GCs was detected using an Annexin V-FITC Apoptosis Detection Kit (KeyGen, Nanjing, China, Cat#KGA106) as the manufacturer’s instructions. Briefly, the cells were seeded in 6-well plates and transfected for 48 h on 80% GC confluence. Then, the cells were washed with cold PBS three times and centrifuged. Next, the cells were resuspended in 500 µL of binding buffer and stained with Annexin V-FITC and Propidium Iodide (PI) staining solution according to the manufacturer’s protocols. The stained cells were analyzed using a FACS Calibur flow cytometer (Beckman Coulter, Miami, FL, USA).

### 2.7. Cell Cycle Assay

The cell cycle of GCs was examined with a cell cycle detection kit (KeyGen, Nanjing, China, Cat#KGA512). The cells were seeded in 6-well plates and transfected for 48 h on 80% GC confluence. Then, the cells were trypsinized and washed with cold PBS three times. After collecting the cells, 500 µL of 70% ethanol was added and the mixture was incubated at 4 °C overnight. Subsequently, all fixative was removed with PBS and the cells were stained with PI/RNase A solution according to the manufacturer’s instructions. The stained cells were analyzed using a FACS Calibur flow cytometer.

### 2.8. Immunofluorescence Staining

When the cells reached approximately 90% confluence in 12-well plates, the culture medium was discarded and the cells were washed three times with PBS and fixed with 4% paraformaldehyde (Biosharp, Hefei, China, Cat#BL539A) for 15 min at room temperature. Then, permeabilization was carried out with 0.1% Triton-X (BioProx, Levallois-Perret, France, Cat#1139ML500) for 15 min followed by blocking with 5% BSA (Servicebio, Wuhan, China, Cat#G5001) for 1 h. Then, the cells were incubated with primary antibodies against IL-11, IL-11Rα, gp130, FSHR, and CYP17A1 (Table S1) overnight at 4 °C. Afterward, the cells were washed with PBS to remove unbound antibodies and incubated with the corresponding secondary antibodies (Table S1) for 1 h at room temperature. Subsequently, the cells were washed and stained with DAPI (Coolaber, Beijing, China, Cat#SL7100). Finally, the cells were sealed with Antifade Mounting Medium (Servicebio, Wuhan, China, Cat#G1401) and images were captured using a Zeiss LSM 800 confocal laser-scanning microscope (Zeiss, Oberkochen, Germany).

### 2.9. Immunohistochemistry Staining

The ovary tissues were washed with PBS and soaked in 4% paraformaldehyde for more than 24 h, dehydrated with ethanol, and embedded into paraffin blocks. The paraffin blocks were cooled in the freezer, sliced into 4 μm thick slices, and mounted on adhesion-treated slides. After deparaffinization, xylene was removed with a graded series of alcohols. Next, the slides were placed into a citric acid buffer (PH 6.0) for antigen retrieval in a microwave and endogenous peroxidase was blocked by a 3% hydrogen peroxide solution (Sinopharm, Beijing, China, Cat#10011208). After blocking with 3% BSA to decrease background staining, the treatment sections were incubated with primary antibodies against IL-11 and IL-11Rα (Table S1) and the negative controls were incubated with 3% BSA overnight at 4 °C. After three washes in PBS, the sections were incubated with the appropriate secondary antibodies (Table S1) for 50 min at room temperature and then stained with 3,3′-diaminobenzidine (DAB) solution (Servicebio, Wuhan, China, Cat#G1212). Subsequently, the sections were restained with hematoxylin (Servicebio, Wuhan, China, Cat#G1004) for 3 min and then dehydrated and sealed. Images were observed and captured under a microscope (Nikon, Tokyo, Japan).

### 2.10. Hormone Detection

The follicular fluid (FF) from large (≥10 mm), medium and small follicles (2–9.9 mm) of healthy cows were collected and the concentrations of IL-11 in the diluted follicular fluid were detected by the bovine interleukin 11 (IL-11) ELISA kit (NecErice, Wuhan, China, Cat#FB-XZ77266). The cells were seeded in 12-well plates and treated with 0 or 10 μM of FSK for 48 h when the cells reached 80% confluence. Progesterone (P4) and estradiol (E2) in the diluted culture medium of GCs were measured using the Bovine Estradiol (E2) ELISA kit (NecErice, Wuhan, China, Cat#FB-XZ77039) and Bovine Progesterone (PROG) ELISA kit (CUSABIO, Wuhan, China, Cat#CSB-E08172b) according to the manufacturer’s protocols. Finally, the absorbance at 450 nm was measured using a microplate reader. The concentrations of IL-11, E2, and P4 were established according to the corresponding standard curve. The ranges of the standard curves were 7.5–120 pg/mL for IL-11, 1.6–80 pg/mL for E2, and 0.15–70 ng/mL for P4. The intra- and inter-assay coefficients of variation were less than 10.0% and 15.0% for IL-11, 15.0% and 15.0% for E2, and 15.0% and 15.0% for P4. The E2 and P4 concentrations were normalized by the corresponding cellular protein concentrations.

### 2.11. Quantitative Real-Time PCR (qRT-PCR) Assay

The tissues were washed with DEPC and homogenized using grinding beads. The GCs were cultured and transfected with IL-11Rα siRNA or siRNA-NC for 48 h. Total RNA was extracted from tissues and GCs using the E.Z.N.A. ^®^ Total RNA Kit II (OMEGA Bio-Tek, Norcross, GA, USA, Cat#R6934-01) according to the manufacturer’s protocols, and the RNA concentrations were quantified using a NanoDrop^TM^ 2000 spectrophotometer (Thermo Fisher Scientific, Waltham, MA, USA). For cDNA synthesis, 1 μg of total RNA was used to generate cDNA by HiScript II Q RT SuperMix (Vazyme, Nanjing, China, Cat#R223-01). The qRT-PCR was performed with a ChamQ Universal SYBR qPCR Master Mix (Vazyme, Nanjing, China, Cat#Q711-02) on a CFX384 real-time PCR detection system (Bio-Rad, Hercules, CA, USA). Specific primers were designed using Primer Premier 5.0, as shown in Table S2. Each 10 μL of reaction solution was composed of 5 μL SYBR Green Mix, 0.2 μL each of both forward and reverse primers, 3.6 μL H_2_O, and 1 μL cDNA. All samples were run under the following conditions: 95 °C for 2 min, 40 cycles of amplifications (95 °C for 10 s, 60 °C for 30 s, and 72 °C for 30 s). Melting curve analysis was performed in the range of 65 to 95 °C with an increase of 0.5 °C per 5 s. The relative expression of genes was calculated using the 2^−ΔΔCT^ method and normalized with the reference gene GAPDH.

### 2.12. Western Blotting Analysis

The cells were seeded in 6-well plates and transfected with IL-11Rα siRNA or siRNA-NC for 24 and 48 h when the cells reached 80% confluence. GCs were lysed for 30 min on ice with a RIPA lysis buffer (Servicebio, Wuhan, China, Cat#G2002) containing 1% PMSF (Servicebio, Wuhan, China, Cat#G2008), 2% cocktail (Servicebio, Wuhan, China, Cat#G2006), 1% phosphatase inhibitor A solution (Servicebio, Wuhan, China, Cat#G2007-1), and 1% phosphatase inhibitor B solution (Servicebio, Wuhan, China, Cat#G2007-2). The collected cell lysate was boiled for 5 min with a protein loading buffer (Servicebio, Wuhan, China, Cat#G2013) to denature the protein. Afterward, 20 µg of cell protein was separated using 10% sodium dodecyl sulfate-polyacrylamide gel electrophoresis (SDS-PAGE) and transferred onto a 0.22 µm polyvinylidene fluoride (PVDF) membrane (Epizyme, Shanghai, China, Cat#WJ001). After being blocked with 1×blocking buffer (Epizyme, Shanghai, China, Cat#PS108P) for 1 h at room temperature, the membranes were incubated at 4 °C overnight with the corresponding primary antibodies against IL-11Rα, PCNA, BAX, Cyclin D1, JAK1, phospho-JAK1, JAK2, phospho-JAK2, STAT3, phospho-STAT3, mTOR, phospho-mTOR, CYP19A1, StAR, phospho-PKA, CREB, phospho-CREB, p38MAPK, phospho-p38MAPK, phospho-ERK1/2, ERK1/2, and GAPDH (Table S1). These membranes were washed three times with TBST and incubated using horseradish peroxidase (HRP)-conjugated secondary antibody (Table S1) for 2 h at room temperature. Finally, the membranes were washed three times and developed with an enhanced chemiluminescence (ECL) kit (Biosharp, Hefei, China, Cat#BL520A) using a chemiluminescence imaging system (Tanon, Shanghai, China). The grayscale scanning results of the Western blotting were analyzed using Image J software (version 1.8.0, NIH). 

### 2.13. Statistical Analysis

Three independent experiments were conducted in this study unless otherwise stated. GraphPad 6.0 software was used for the analysis of the results. The data were analyzed using the Student’s *t*-test and are presented as the mean ± standard error of the mean (SEM). Differences were considered significant at (*) or (^#^) *p* < 0.05, (**) or (^##^) *p* < 0.01.

## 3. Results

### 3.1. Tissue Differential Expression of IL-11 and IL-11Rα in Bovine Organs 

**Figure 1:**
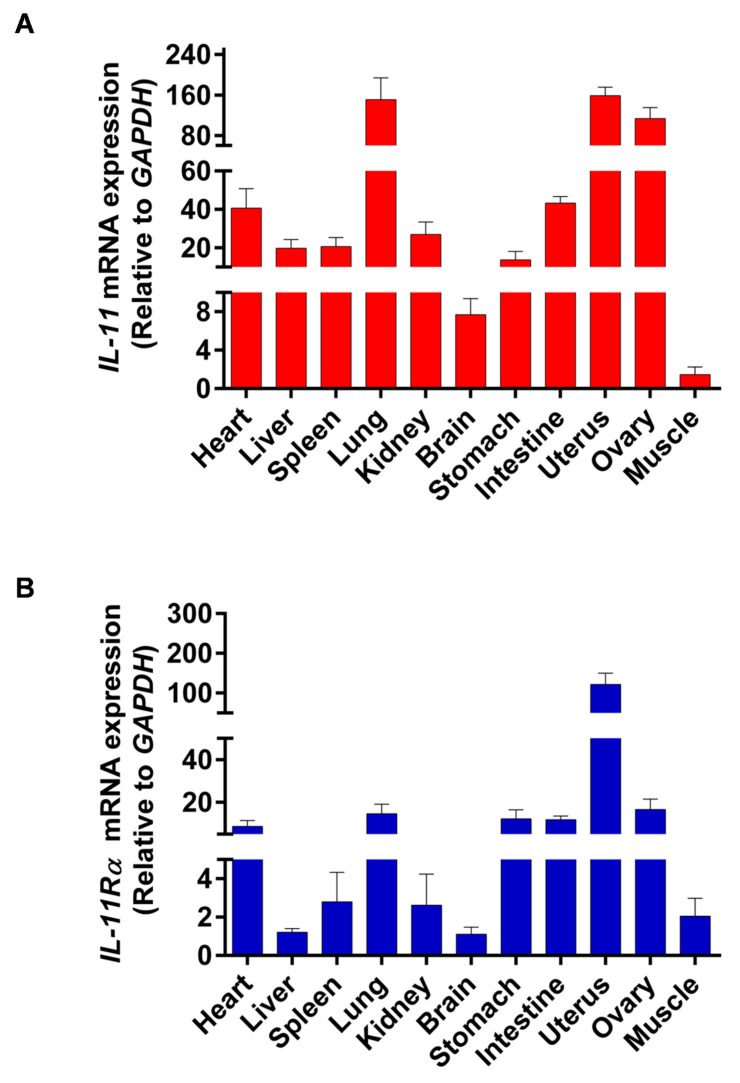
Tissue distribution of IL-11 and IL-11Rα in bovine organs. (**A**) The relative mRNA expression levels of IL-11 were quantitated in various tissues by qRT-PCR and normalized by GAPDH. The values are shown as the fold change above the muscle tissue (mean Cq value of the muscle is 27.46 and set to 1). (**B**) The relative mRNA expression levels of IL-11Rα were quantitated in various tissues by qRT-PCR and normalized by GAPDH. The values are shown as the fold change above the brain tissue (mean Cq value of the brain is 23.60 and set to 1).

The tissue distribution of *IL-11* and *IL-11Rα* mRNA expression was analyzed by qRT-PCR in all of the investigated tissues, including the heart, liver, spleen, lung, kidney, brain, stomach, intestine, uterus, ovary, and muscle. We found that the uterus showed the highest *IL-11* mRNA expression, followed by the lung, ovary, intestine, and heart, whereas the muscle tissue had the lowest *IL-11* mRNA level (Figure 1A). Similarly, the uterus exhibited the highest expression of *IL-11Rα* mRNA, followed by the ovary, lung, stomach, intestine, and heart (Figure 1B). The high expression of *IL-11* and *IL-11Rα* in the ovary indicated that the IL-11 system may play a crucial role in ovarian function.

### 3.2. Localization of IL-11 System in Ovary and IL-11 Concentration in FF 

**Figure 2:**
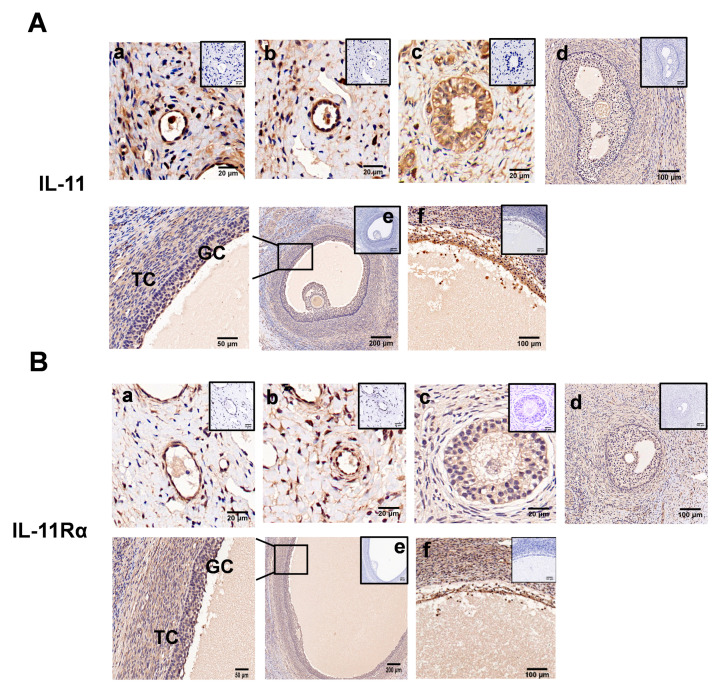
Immunohistochemical staining of IL-11 and IL-11Rα in bovine ovary. IL-11 (**A**) and IL-11Rα (**B**) were detected in primordial (**a**); a single layer of flattened GCs, primary (**b**); a single layer of cuboidal GCs, secondary (**c**); multiple layers of cuboidal GCs, small antral (**d**); large antral (**e**); and atretic follicles (**f**). GC, granulosa cells; TC, theca cells. Negative controls were incubated in the absence of the primary antibody and are presented in the top-right corner. Scale bars correspond to 20 (**a**–**c**), 50 (enlarged **e**), 100 (**d**,**f**), and 200 μm (**e**).

Immunohistochemical analysis showed that both IL-11 and IL-11Rα were present in the primordial (Figure 2A,B, panel a), primary (Figure 2A,B, panel b), secondary (Figure 2A,B, panel c), and antral follicles (Figure 2A,B, panel d,e), and more precisely in the GCs, theca cells, and oocytes (Figure 2A,B). In the antral follicles, immunoreactivity was mainly detected in the GCs, although it was also observed at lower levels in the theca cells (TCs). Notably, strong staining was observed in the atretic follicles with a few layers of GCs and some pyknotic nuclei (Figure 2A,B, panel f). No staining was shown in the control tissue sections incubated in the absence of the primary antibody.

**Figure 3:**
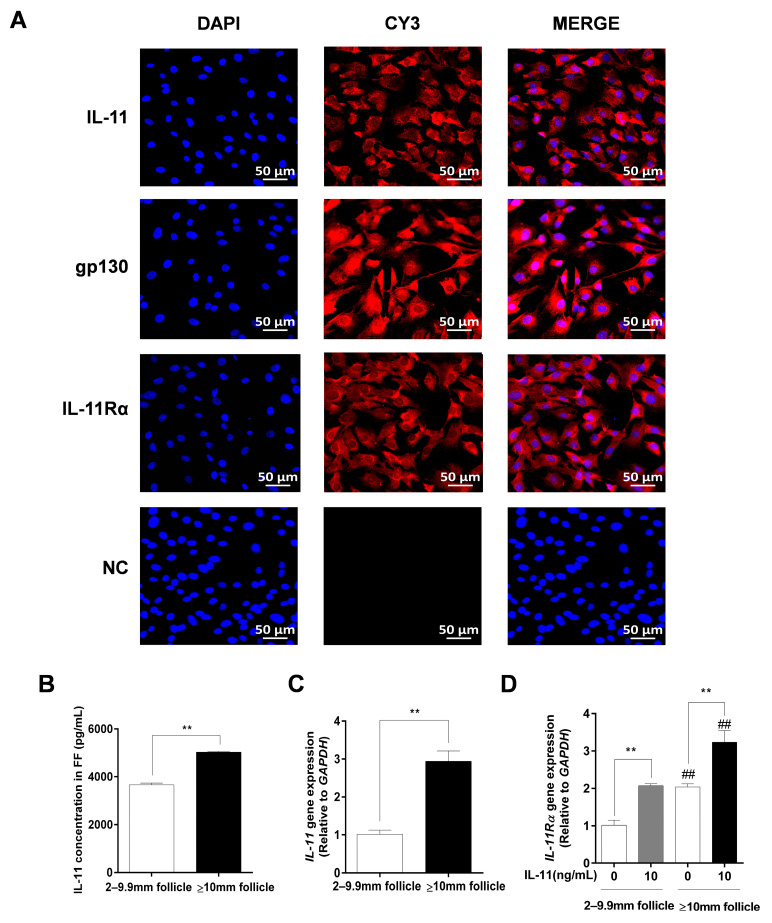
The subcellular localization and expression of the IL-11 system in bovine GCs. (**A**) Cells were stained with specific antibodies against IL-11 (red), gp130 (red), and IL-11Rα (red), and the nucleus was stained with DAPI (blue). The specific signals were examined using indirect immunofluorescence and confocal microscopy. The scale bar represents 50 μm. (**B**) The concentrations of IL-11 in different diameter follicles were measured by ELISA. (**C**) The relative mRNA expression levels of IL-11 were determined in GCs from different diameter follicles by qRT-PCR and normalized by GAPDH. (**D**) The relative mRNA expression levels of IL-11 Rα were detected in GCs from different diameter follicles in the absence or presence of IL-11 by qRT-PCR and normalized by GAPDH. ** *p* < 0.01 between the indicated groups. ^##^ *p* < 0.01 between the control groups or the IL-11 treatment groups.

Immunofluorescence staining further indicated that IL-11, IL-11Rα, and gp130 exhibited strong positive signals in the cytoplasm of the GCs, whereas no fluorescence signal was observed in any of the control groups (Figure 3A). Additionally, we found that the IL-11 concentration in follicular fluid was significantly higher in large follicles (≥10 mm) than in small and medium follicles (2–9.9 mm follicles, *p* < 0.01, Figure 3B). In addition, the mRNA expression levels of *IL-11* and *IL-11Rα* were higher in the GCs of large follicles than those in small and medium follicles (*p* < 0.01, Figure 3C,D). Notably, the addition of 10 ng/mL of IL-11 in bovine GCs resulted in a significant upregulation of *IL-11Rα* mRNA expression in small and medium follicles, as well as in large follicles (*p* < 0.01, Figure 3D).

### 3.3. Detection of Knockdown Efficiency of IL-11Rα in Bovine GCs

**Figure 4:**
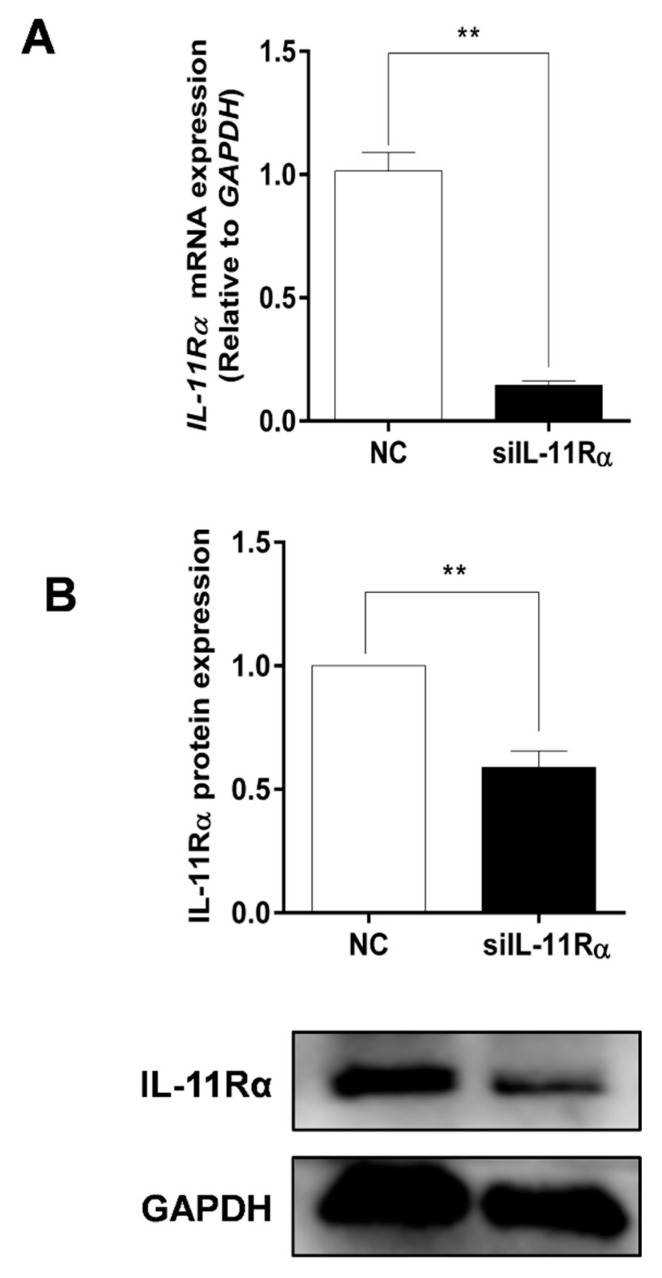
Detection of the efficiency of IL-11Rα knockdown in bovine GCs. Cells were transfected for 48 h with siIL-11Rα and a negative control (NC) siRNA. (**A**) The relative mRNA levels of IL-11Rα were detected by qRT-PCR and normalized by GAPDH. (**B**) The protein levels of IL-11Rα were determined by Western blot and the quantitative results indicated fold changes in the IL-11Rα/GAPDH ratio; the representative Western blots from three independent experiments are shown. ** *p* < 0.01, compared to the control group.

Given the higher expression and secretion of IL-11 in the large follicles, the primary GCs of the large follicles were cultured in subsequent experiments to determine the function of the IL-11 system. Firstly, the purity of the primary GCs was verified with immunofluorescence staining using a GC-specific marker FSHR, whereas the TC-specific marker CYP17A1 was used for reverse identification (Figure S1). Subsequently, the IL-11Rα siRNA was transfected into the GCs and the interference efficiency was assessed. The results showed that the silencing of IL-11Rα markedly decreased mRNA levels by 85.64% (Figure 4A, *p* < 0.01) and protein levels by 41.05% (Figure 4B, *p* < 0.01), suggesting that the GC model of IL-11Rα knockdown was well-established.

### 3.4. Effect of IL-11/IL-11Rα on Cell Proliferation and Apoptosis in Bovine GCs 

**Figure 5:**
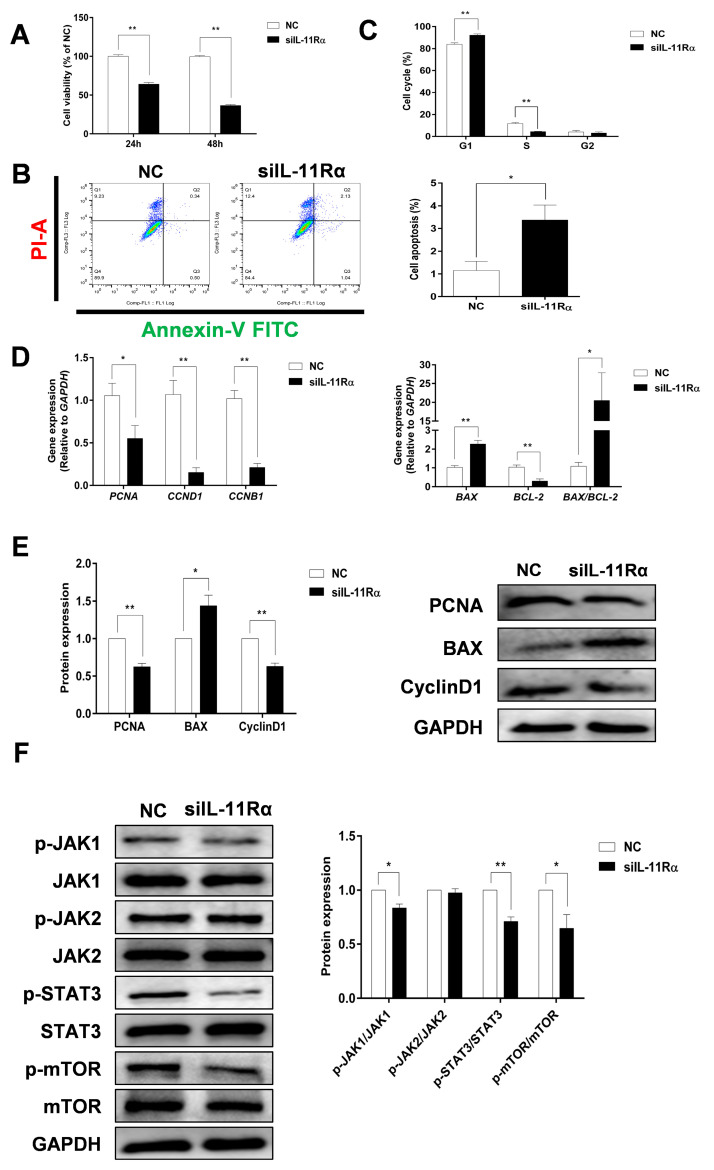
The effect of IL-11Rα knockdown on cell proliferation and apoptosis by bovine GCs. (**A**) After siIL-11Rα transfection for 24 and 48 h, cell viability was determined using the CCK-8 method and the cell viability of the control group was set at 100%, data represent two independent experiments, each completed in triplicate. (**B**,**C**) After siIL-11Rα transfection for 48 h, cell apoptosis was determined by flow cytometry and the cell cycle was assayed by flow cytometry, data represent two independent experiments, each completed in triplicate. (**D**) After siIL-11Rα transfection for 48 h, the relative mRNA expression levels of *PCNA*, *CCND1*, *CCNB1*, *BAX*, *BCL-2*, and the *BAX/BCL-2* ratio were analyzed by qRT-PCR and normalized by GAPDH, data represent two independent experiments, each completed in triplicate. (**E**) The protein expression levels of PCNA, BAX, and Cyclin D1 were detected by Western blot and the quantitative results showed fold changes in PCNA/GAPDH, BAX/GAPDH, and the Cyclin D1/GAPDH ratios. (**F**) The protein levels of phospho-JAK1, JAK1, phospho-JAK2, JAK2, phospho-STAT3, STAT3, phospho-mTOR, and mTOR were detected by Western blot and the quantitative results indicated fold changes in the p-JAK1/JAK1, p-JAK2/JAK2, p-STAT3/STAT3, and p-mTOR/mTOR ratios compared to the control; the representative Western blots from three independent experiments are shown. * *p* < 0.05, ** *p* < 0.01, compared to the control group.

Compared to the negative control (Figure 5A), the cell viability in the siIL-11Rα group significantly decreased by 34.85% and 64.15% after transfection for 24 and 48 h, respectively. IL-11Rα knockdown markedly induced cell apoptosis (Figure 5B, *p* < 0.05). Moreover, the flow cytometry analysis demonstrated that the ratio of cells in the G1 phase increased, whereas the ratio of cells in the S phase decreased (Figure 5C, *p* < 0.01), suggesting that the cell cycle was arrested at the G1 phase. In addition, we observed a significant downregulation in the mRNA expression levels of proliferating cell nuclear antigen (*PCNA)*, Cyclin B1 (*CCNB1*), Cyclin D1 (*CCND1*), and *BCL-2* genes after IL-11Rα silencing and a significant upregulation in the mRNA expression of *BCL-2*-associated X (*BAX)* and the ratio of *BAX/BCL-2* (Figure 5D). Western blot analysis further indicated that the protein expression of PCNA and Cyclin D1 significantly decreased after IL-11Rα knockdown, whereas the protein expression of BAX increased (Figure 5E).

We carried out an additional investigation into the downstream signaling pathway related to the anti-apoptotic effect of the IL-11 system in GCs. As shown in Figure 5F, IL-11Rα silencing significantly decreased the phosphorylation of JAK1 at Tyr^1034/1035^, STAT3 at Tyr^705^, and mTOR at Ser^2448^ compared to the negative control. In contrast, neither the phosphorylated nor total protein of JAK2 was affected after the suppression of IL-11Rα expression. The data revealed that IL-11Rα knockdown inhibited GC proliferation and induced apoptosis by decreasing the activity of the JAK1/STAT3 signaling pathway.

Additionally, we investigated the effects of IL-11 on proliferation and apoptosis by supplementing with 10 ng/mL of IL-11 in bovine GCs. Compared to the control group, we found that 10 ng/mL of IL-11 significantly increased the cell viability (*p* < 0.01), accompanied by an upregulation in mRNA expression levels of *PCNA*, *CCNB1*, and *CCND1* (Figure S2A,B). In contrast, the addition of IL-11 in bovine GCs significantly reduced cell apoptosis (*p* < 0.01), along with the upregulation of the *BCL-2* gene and the downregulation of *BAX* and *BAX/BCL-2* (Figure S2C–E).

### 3.5. Effect of IL-11Rα Knockdown on Steroidogenesis in Bovine GCs

**Figure 6:**
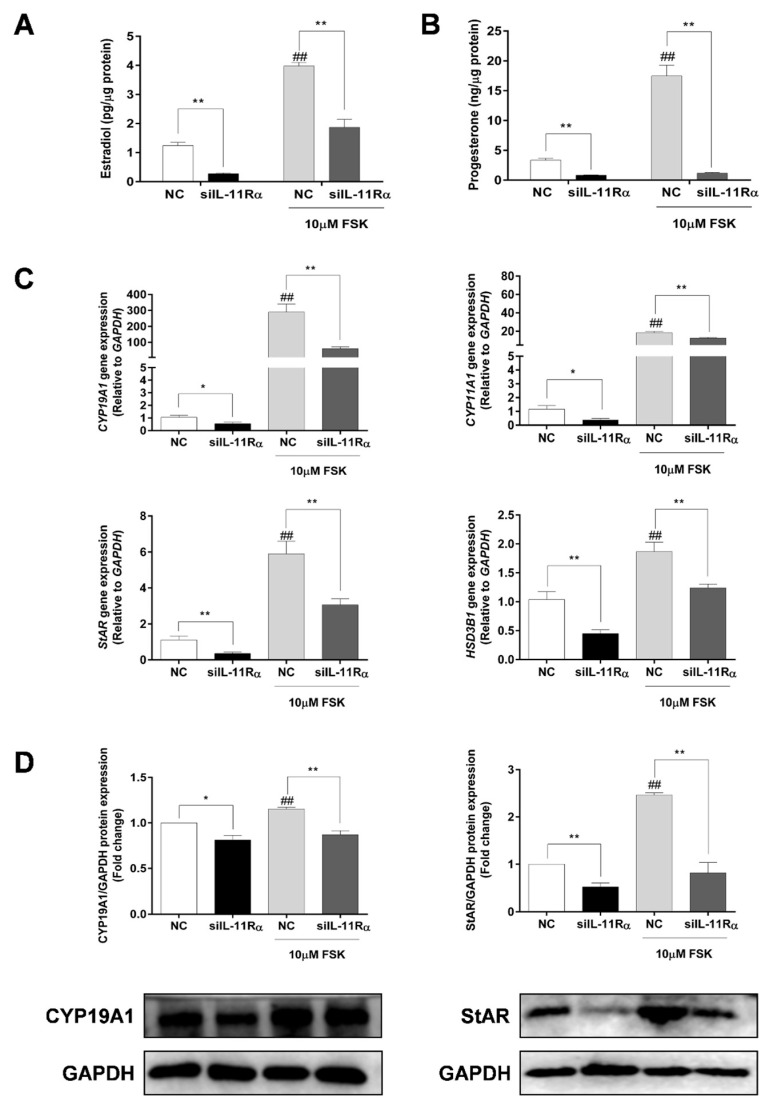
Effect of IL-11Rα knockdown on steroidogenesis by bovine GCs after 48 h of siIL-11Rα transfection. Estradiol (**A**) and progesterone (**B**) production in the presence or absence of 10 μM FSK were determined by ELISA; data represent two independent experiments, each completed in triplicate. (**C**) The relative mRNA expression levels of *CYP19A1*, *CYP11A1*, *StAR*, and *HSD3B* transcripts in the presence or absence of 10 μM FSK and normalized by GAPDH; data represent two independent experiments, each completed in triplicate. (**D**) The protein levels of CYP19A1 and StAR in the presence or absence of 10 μM FSK were examined by Western blot; the quantitative results indicated fold changes in the CYP19A1/GAPDH and StAR/GAPDH ratios compared to the control and the representative Western blots from three independent experiments are shown. * *p* < 0.05 and ** *p* < 0.01 between the indicated groups. ^##^ *p* < 0.01 between the negative control (-FSK) and the FSK treatment-alone groups.

Considering that FSK (adenylyl cyclase activator) is a well-known factor in the stimulation of steroidogenesis in GCs, we further compared the effects of IL-11Rα on steroidogenesis in the presence or absence of FSK. Expectedly, the FSK treatment significantly increased both estrogen and progesterone productions by 3.19- and 5.22-fold (Figure 6A,B, *p* < 0.01 for both) compared to the basal state. Importantly, IL-11Rα knockdown not only decreased the basal secretion of estradiol and progesterone by 78.15% and 75.26% but also attenuated the FSK-induced secretion of these hormones (Figure 6A,B, *p* < 0.01). Furthermore, the qRT-PCR results showed that IL-11Rα knockdown significantly suppressed the basal and FSK-induced mRNA expression of cytochrome P450 family 19 subfamily A member 1 (*CYP19A1*), which is a key enzyme for estrogen biosynthesis. Similarly, IL-11Rα knockdown decreased the basal and FSK-stimulated mRNA expression of cytochrome P450 family 11 subfamily A member 1 (*CYP11A1*), steroidogenic acute regulatory protein (*StAR*), and 3 beta-hydroxysteroid dehydrogenase/Delta (5)-Delta (4) isomerase (*HSD3B*), which are related to progesterone production (Figure 6C). The Western blot analysis also indicated a significant decrease in the protein expression of CYP19A1 and StAR at both the basal and FSK-induced levels in the IL-11Rα knockdown group (Figure 6D). These data demonstrated that IL-11Rα silencing reduced steroidogenesis by downregulating the expression of steroidogenic-related genes. 

### 3.6. Involvement of PKA Signaling in Steroidogenesis after IL-11Rα Knockdown

**Figure 7:**
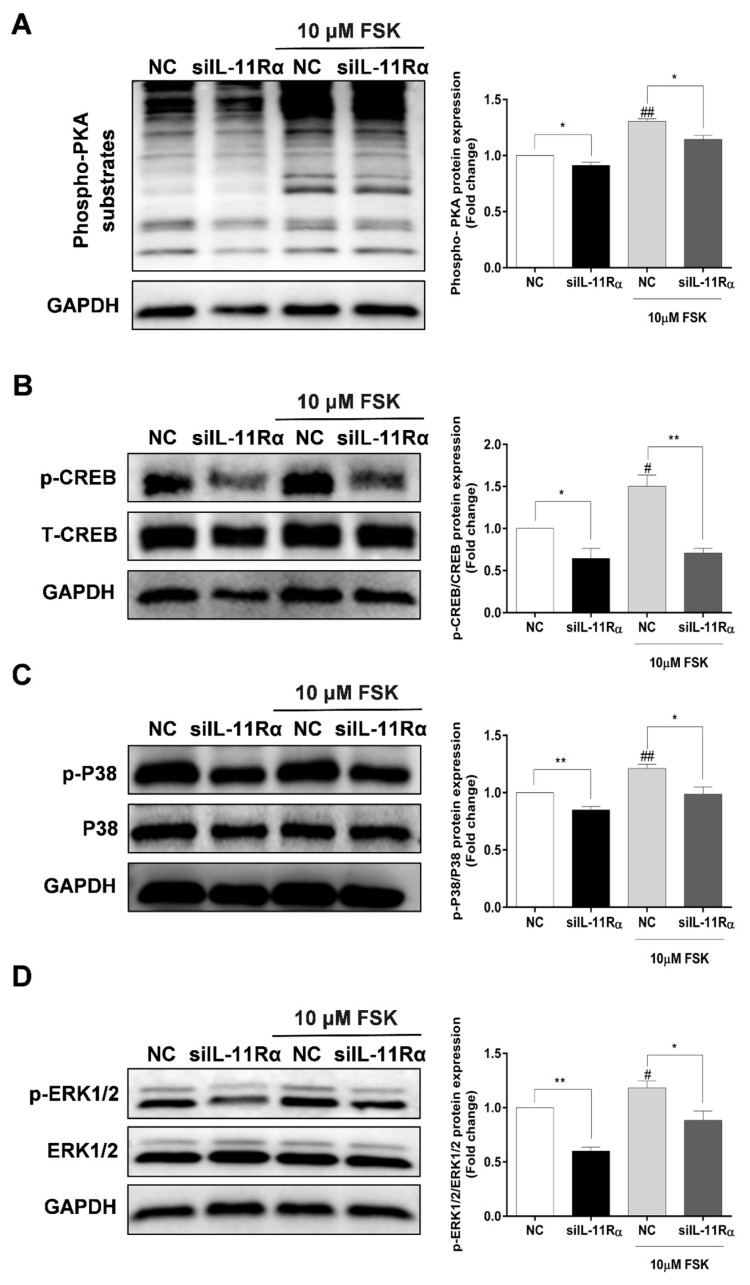
Effect of IL-11Rα knockdown on the phosphorylation of PKA substrates, CREB, p38MAPK, and ERK1/2. (**A**) After 48 h of siIL-11Rα transfection, the protein levels of phospho-PKA substrates were examined by Western blot, the quantitative results indicated fold change in the phospho-PKA/GAPDH ratio compared to the negative control. (**B**) After 48 h of siIL-11Rα transfection, the quantitative results indicated fold change in the phospho-CREB/CREB ratio compared to the negative control. (**C**) After 48 h of siIL-11Rα transfection, the quantitative results indicated fold change in the phospho-p38MAPK/p38MAPK ratio compared to the negative control. (**D**) After 48 h of siIL-11Rα transfection, the quantitative results indicated fold change in the phospho-ERK/ERK ratio compared to the negative control. The representative Western blots from three independent experiments are shown. * *p* < 0.05 and ** *p* < 0.01 between the indicated groups. ^#^ *p* < 0.05 and ^##^ *p* < 0.01 between the negative control (-FSK) and the FSK treatment-alone groups.

As shown in Figure 7A, IL-11Rα knockdown significantly decreased the phosphorylation of PKA substrates at the basal level. As expected, we observed a significant enhancement of the phosphorylation of PKA substrates in the presence of FSK and this induction was significantly inhibited by IL-11Rα knockdown. Subsequently, we detected the downstream effectors of the PKA pathway and observed that FSK stimulated the phosphorylation of CREB at Ser^133^, p38 at Thr^180^/Tyr^182^, and ERK1/2 at Thr^202^/Tyr^204^, whereas IL-11Rα knockdown decreased the phosphorylation of CREB at Ser^133^, p38 at Thr^180^/Tyr^182^, and ERK1/2 at Thr^202^/Tyr^204^ (Figure 7B–D). These findings suggest that the IL-11 system regulates the phosphorylation of CREB in GCs through the PKA-CREB, p38MAPK, and ERK1/2 pathways. 

## 4. Discussion

IL-11 is a pleiotropic cytokine and its responsiveness relies on the cells that express the IL-11Rα and gp130 receptors. Because the shared gp130 subunit is ubiquitously present, the cellular response to IL-11 is determined by the more restricted expression pattern of IL-11Rα [7,26,27]. Before studying the effects of the IL-11 system on proliferation and steroidogenesis, we first detected the mRNA expression of the IL-11 system in bovine tissues and found that the uterus exhibited the highest expression of IL-11 and IL-11Rα, which is associated with their crucial roles in the decidualization of the uterus [9,11]. Furthermore, we demonstrated that IL-11 and IL-11Rα were highly expressed in bovine GCs at different developmental stages, as previously observed in rats [23]. The level of IL-11 was found to be the highest in large follicles, similar to a previous study showing higher secretion of IL-11 in human follicular fluid [22], indicating its potential role as a contributory factor in the ovulatory process. Notably, we also observed that atretic follicles displayed higher expression of IL-11 and IL-11Rα, which is consistent with a previous report that showed that human atretic follicles had higher concentrations of IL-11 [22]. However, IL-11 is rarely detected in the serum of healthy individuals [22], suggesting that IL-11 exerts biological activity in the ovaries as an autocrine or paracrine factor.

The proliferation and apoptosis of GCs affect follicular development and ovulation. Besides its well-known anti-inflammatory activity, IL-11 has been shown to have anti-apoptotic properties in the colonic epithelium [7], intestinal cells [28], and hepatocytes [29]. In this study, we observed that IL-11Rα knockdown promoted cell apoptosis, which confirmed its anti-apoptotic effect in bovine GCs. In addition, we revealed that the anti-apoptotic gene BCL-2 significantly decreased, whereas the pro-apoptotic gene BAX significantly increased after IL-11Rα knockdown. The ratio of BAX/BCL-2 significantly increased, indicating that BAX and BCL-2 synergistically regulate the apoptosis of bovine GCs transfected with IL-11Rα siRNA. On the other hand, our findings indicated that supplementation with IL-11 in bovine GCs had pro-proliferative and anti-apoptotic effects. Furthermore, we found that IL-11Rα knockdown inhibited cell proliferation through cell cycle arrest in the G1 phase. Importantly, we demonstrated the effect of IL-11Rα silencing on proliferation and the cell cycle by downregulating the expression of PCNA, CyclinD1, and CyclinB1, which are involved in the regulation of proliferation and the cell cycle in GCs [30,31]. An important signaling system activated by the IL-11 system is the JAK-STAT3 pathway [13,32], which is related to cell proliferation and survival. Numerous studies have demonstrated that the activation of STAT3 could promote the G1/S phase transition, as well as prevent apoptosis by regulating the expression of certain genes such as Cyclin D1, Cyclin B1, and BCL-2 [10,33,34]. Here, we revealed that IL-11Rα knockdown attenuated the phosphorylation of JAK1 but not JAK2 and, subsequently, decreased the phosphorylation of STAT3, suggesting that JAK1 preferentially activates STAT3 phosphorylation through the IL-11 system. Our results confirmed the previous findings of Shien et al. [35], showing that the JAK family of tyrosine-kinases, especially JAK1, is a critical type of kinase that mediates the activation of STAT3 through IL-6 cytokines in NSCLC cells.

The steroid hormones synthesized by GCs play an important role in the regulation of follicular development. As a member of the IL-6 family of cytokines, IL-6 has been widely investigated and was shown to be involved in the regulation of estradiol and progesterone secretion in GCs of humans [36,37], rats [38], pigs [39], and cattles [40]. More precisely, IL-6 exerted a significant inhibitory role in FSH-stimulated estradiol and progesterone secretion, as well as hCG-driven progesterone secretion by human GCs [36,37]. Similarly, IL-6 significantly inhibited FSH-induced estradiol production by 32% in the bovine GCs of large follicles [40]. However, IL-6 has been reported to stimulate LH-induced progesterone levels but not estradiol in rats [38], which has led to divisive findings. Regarding IL-11, one study demonstrated that IL-11 increased progesterone production in preovulatory follicles of rats but had no effect on estradiol production [23]. In the present study, we provided evidence that IL-11Rα knockdown led to a significant reduction in basal and FSK-induced progesterone production, which further confirmed the stimulatory effect of IL-11 on progesterone levels [23]. Nevertheless, we also demonstrated that IL-11Rα knockdown inhibited basal and FSK-stimulated estradiol secretion, the discrepant observation on estradiol may be due to the different species and the cell sources. Furthermore, our results are supported by the downregulation of the expression of genes related to steroid biosynthesis, including CYP19A1, StAR, CYP11A1, and HSD3B. Taken together, our results showed that IL-11Rα knockdown decreased basal and FSK-induced estradiol and progesterone production, indicating that IL-11 and IL-6 may exert different effects on steroidogenesis despite the fact that they share the common receptor of gp130. 

Accumulating evidence has demonstrated that cAMP-PKA-CREB signaling is the classic pathway modulating steroidogenesis by GCs and the activation of the CREB transcription factor is involved in the regulation of steroidogenic enzymes such as StAR and CYP19A1 [41,42]. In the present study, we showed that FSK significantly stimulated the phosphorylation of PKA substrates and subsequently activated the phosphorylation of CREB, which is consistent with previous findings in porcine GCs [43]. Importantly, IL-11Rα silencing significantly attenuated the phosphorylation of the PKA substrates and CREB in the absence or presence of FSK, suggesting that the effect of IL-11Rα on steroidogenesis is at least partly mediated by the cAMP-PKA-CREB signaling pathway. In addition to affecting the phosphorylation of CREB, we also demonstrated that IL-11Rα knockdown could inactivate the phosphorylation of p38MAPK and ERK1/2, probably in both a PKA-dependent, as well as an independent manner, and participate in the regulation of CYP19A1 and StAR expression in GCs [42,44,45,46,47]. Notably, the change in CYP19A1 expression may be due to the alteration of p38MAPK or ERK1/2 phosphorylation but not CREB since a site for CREB binding to the bovine aromatase gene is destroyed [48]. Collectively, these findings suggest that the IL-11 system regulates estradiol and progesterone productions in GCs through the PKA-CREB, p38MAPK, and ERK1/2 pathways.

## 5. Conclusions

Our results revealed that IL-11 and IL-11Rα are mainly expressed in large antral follicles. IL-11Rα knockdown inhibited proliferation and promoted apoptosis of bovine GCs by inducing cell cycle arrest at the G1 phase. Moreover, IL-11Rα knockdown decreased basal and FSK-induced estradiol and progesterone productions by downregulating the expression of CYP19A1 and StAR protein. Additional molecular mechanisms showed the effect of IL-11Rα on steroidogenesis in GCs mediated by the PKA-CREB, ERK1/2, and p38MAPK signaling pathways. Our study provides important insights into the autocrine and paracrine actions of the IL-11 system on ovarian function.

## Data Availability

All data generated or analyzed during this study are included in this published article. The original data will be made available on reasonable request.

## Supplementary Materials

The following supporting information can be downloaded at: https://www.mdpi.com/article/10.3390/cells12040673/s1, Figure S1: Purity identification of primary bovine GCs; Figure S2: The effects of IL-11 on the proliferation and apoptosis in bovine GCs; Table S1: The antibodies used in this study; Table S2: The primers used in this study.
