# Supplementary material for: Effects of IL-11/IL-11 Receptor Alpha on Proliferation and Steroidogenesis in Ovarian Granulosa Cells of Dairy Cows
Source: Cells. 2023 Feb 20;12(4):673. doi: 10.3390/cells12040673 (PMC9954560; doi:10.3390/cells12040673)
Supplement: Supplementary file 1 [file cells-12-00673-s001.zip › table s1.pdf]

Table S1 Antibodies used in this study

Antibodies	Dilution ratio	Supplier	RRID	Cat. No	MW（KD）
IL-11Rα	1:1000[1]/1:100[3]	Affinity, USA	AB_2837056	DF4705	45
IL-11Rα	1:200[2]	ABclonal, China	AB_2921269	A9365	
IL-11	1:200[2]/1:100[3]	ABclonal, China	AB_2763933	A1902	
gp130	1:200[2]	ABclonal, China	AB_2861832	A18036	
FSHR	1:200[2]	ABclonal, China	AB_2761677	A1480	
CYP17A1	1:200[2]	Bioss, China	AB_10855146	bs-3853R	
BAX	1:1000[1]	Affinity, USA	AB_2833304	AF0120	21
PCNA	1:1000[1]	Proteintech, USA	AB_2160330	10205-2-AP	36-38
CyclinD1	1:1000[1]	Proteintech, USA	AB_10793718	60186-1-Ig	34
StAR	1:1000[1]	ABclonal, China	AB_2772418	A16432	28
CYP19A1	1:500[1]	Cell Signaling, USA	AB_2630344	14528s	50
CREB	1:1000[1]	Cell Signaling, USA	AB_331277	9197s	43
Phospho-CREB (Ser133)	1:1000[1]	Cell Signaling, USA	AB_2561044	9198s	43
JAK1	1:1000[1]	Cell Signaling, USA	AB_2128499	3332s	130
Phospho-JAK1 (Tyr1034/1035)	1:1000[1]	Cell Signaling, USA	AB_2265057	3331s	130
JAK2	1:1000[1]	Cell Signaling, USA	AB_10691469	4040s	125
Phospho-JAK2 (Tyr1007/1008)	1:1000[1]	Cell Signaling, USA	AB_330403	3771s	125

STAT3	1:1000[1]	Cell Signaling, USA	AB_2629499	12640s	79,86
Phospho-STAT3 (Tyr705)	1:1000[1]	Cell Signaling, USA	AB_2491009	9145s	79,86
mTOR	1:1000[1]	Cell Signaling, USA	AB_2105622	2983T	289
Phospho-mTOR (Ser2448)	1:1000[1]	Abcam, UK	AB_10888105	ab109268	289
p44/42 MAPK (ERK1/2)	1:1000[1]	Cell Signaling, USA	AB_390779	4695s	42,44
Phospho-ERK1/2 (Thr202/Tyr204)	1:1000[1]	Cell Signaling, USA	AB_2315112	4370s	42,44
Phospho-PKA Substrates	1:1000[1]	Cell Signaling, USA	AB_331817	9624s	
p38 MAPK	1:1000[1]	Affinity, USA	AB_2835277	AF6456	43; 41(Calculated)
Phospho-p38MAPK (Thr180/Tyr182)	1:1000[1]	Affinity, USA	AB_2835330	AF4001	43; 41(Calculated)
GAPDH	1:5000[1]	ABclonal, China	AB_2736879	AC002	36
HRP Goat Anti-Mouse IgG (H+L)	1:8000[1]	ABclonal, China	AB_2769851	AS003	
HRP Goat Anti-Rabbit IgG (H+L)	1:8000[1]	ABclonal, China	AB_2769854	AS014	
FITC Goat Anti-Rabbit IgG (H+L)	1:200[2]	ABclonal, China	AB_2769476	AS011	
Cy3 Goat Anti-Rabbit IgG (H+L)	1:200[2]	ABclonal, China	AB_2769089	AS007	
HRP Goat Anti-Rabbit IgG (H+L)	1:200[3]	Servicebio, China	AB_2892100	G1213	

[1]Dilution used for Western blotting.

[2]Dilution used for Immunofluorescence.

[3]Dilution used for Immunohistochemistry.
