# Supplementary material for: Effects of IL-11/IL-11 Receptor Alpha on Proliferation and Steroidogenesis in Ovarian Granulosa Cells of Dairy Cows
Source: Cells. 2023 Feb 20;12(4):673. doi: 10.3390/cells12040673 (PMC9954560; doi:10.3390/cells12040673)
Supplement: Supplementary file 1 [file cells-12-00673-s001.zip › table s2.pdf]

Table S2 Sequences of primers for qRT-PCR

Genes	Sequence (5′-3′)	Length (bp)	Accession no.
GAPDH	GGTCACCAGGGCTGCTTTTA<br>CCAGCATCACCCCACTTGAT	222	**NM_001034034.2**
IL-11Rα	GGCTCTGGCTAAGGTTGAG<br>TGTCCTGCCTGGGTTCTA	183	**NM_001034339.1**
IL-11	ATGAACAGTGTTTGCTGCCTG<br>CTCCGTCAGCTGGGAATTTG	205	**XM_024979371.1**
BAX	TCACCGCCTCGCTCACCATC<br>CCCCCAAGACCACACCTCCC	178	**NM_173894.1**
BCL-2	TTCAGCATCACGGAGGAGGTA<br>TCTTCAATCACGCGGAACACT	144	**NM_001166486.1**
PCNA	GAACCTCACCAGCATGTCCAA<br>TTCACCAGAAGGCATCTTTACT	220	**NM_001034494.1**
CCND1	CCGTCCATGCGGAAGATC<br>CAGGAAGCGGTCCAGGTAG	108	**NM_001046273.2**
CCNB1	GGAAGTTTTGTGTTTTGTGG<br>AGTGGTGGTGATAGAGGAGG	244	**NM_001045872.1**
CYP19A1	ATCGGCATGCACGAGAAAGG<br>CAACACGTCCACATAGCCCA	192	**NM_174305.1**
CYP11A1	TCCAGAACTGTACCGTCTATT<br>GCCTGGGTAATTCCTAAA	145	**NM_176644.2**
StAR	TGATCCCTGACGTGGGC<br>TGAGTGATGACCGTGTCTTTT	169	**NM_174189.3**
HSD3B	CTTGCCGAGAAGGCTGTG<br>TGGTCAGGATGCCGTTG	160	**NM_174343.3**
