# Supplementary figures and images for: Effects of IL-11/IL-11 Receptor Alpha on Proliferation and Steroidogenesis in Ovarian Granulosa Cells of Dairy Cows
Source: Cells. 2023 Feb 20;12(4):673. doi: 10.3390/cells12040673 (PMC9954560; doi:10.3390/cells12040673)

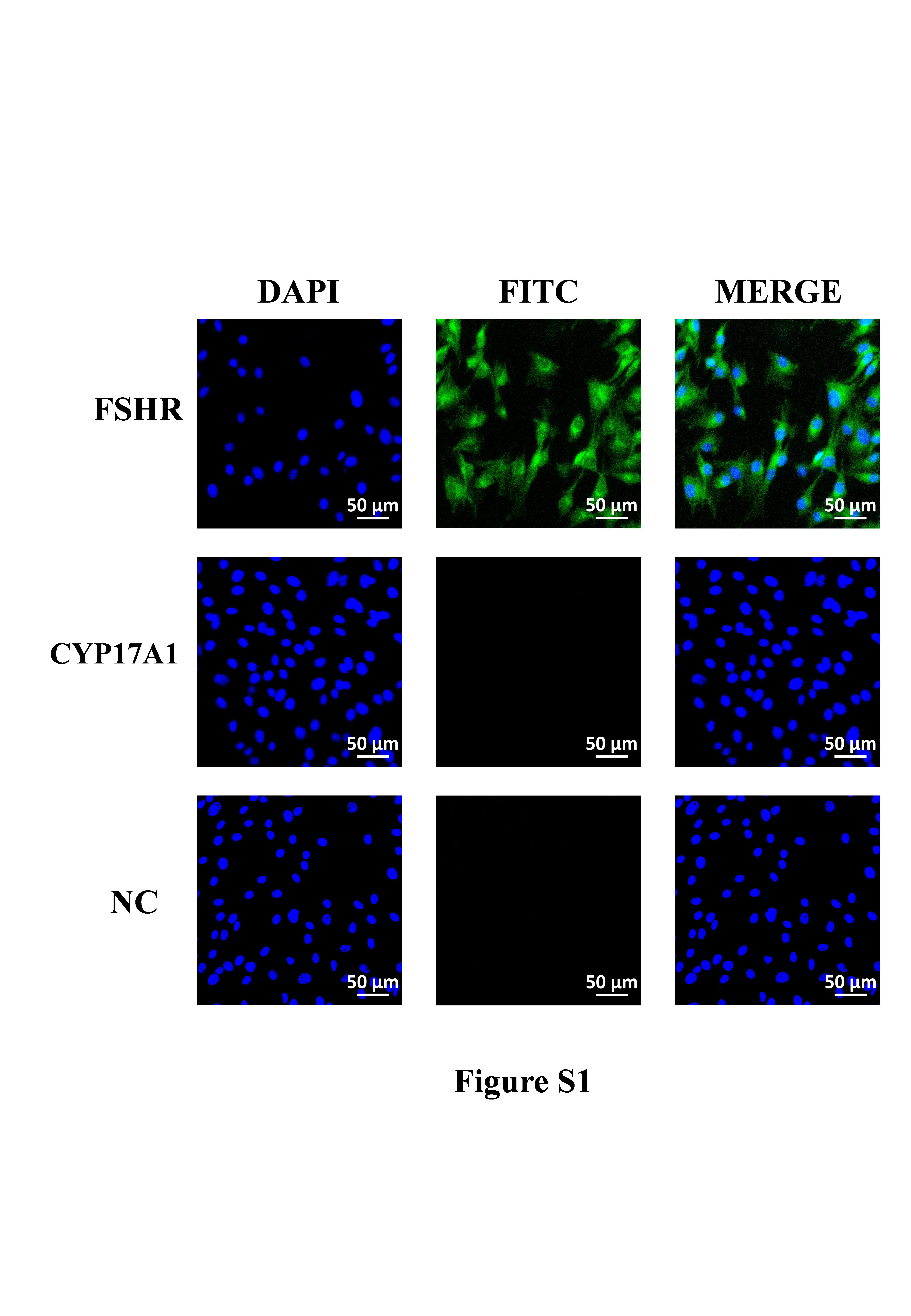

Supplement: Supplementary file 1 [file cells-12-00673-s001.zip › Figure S1.tif]

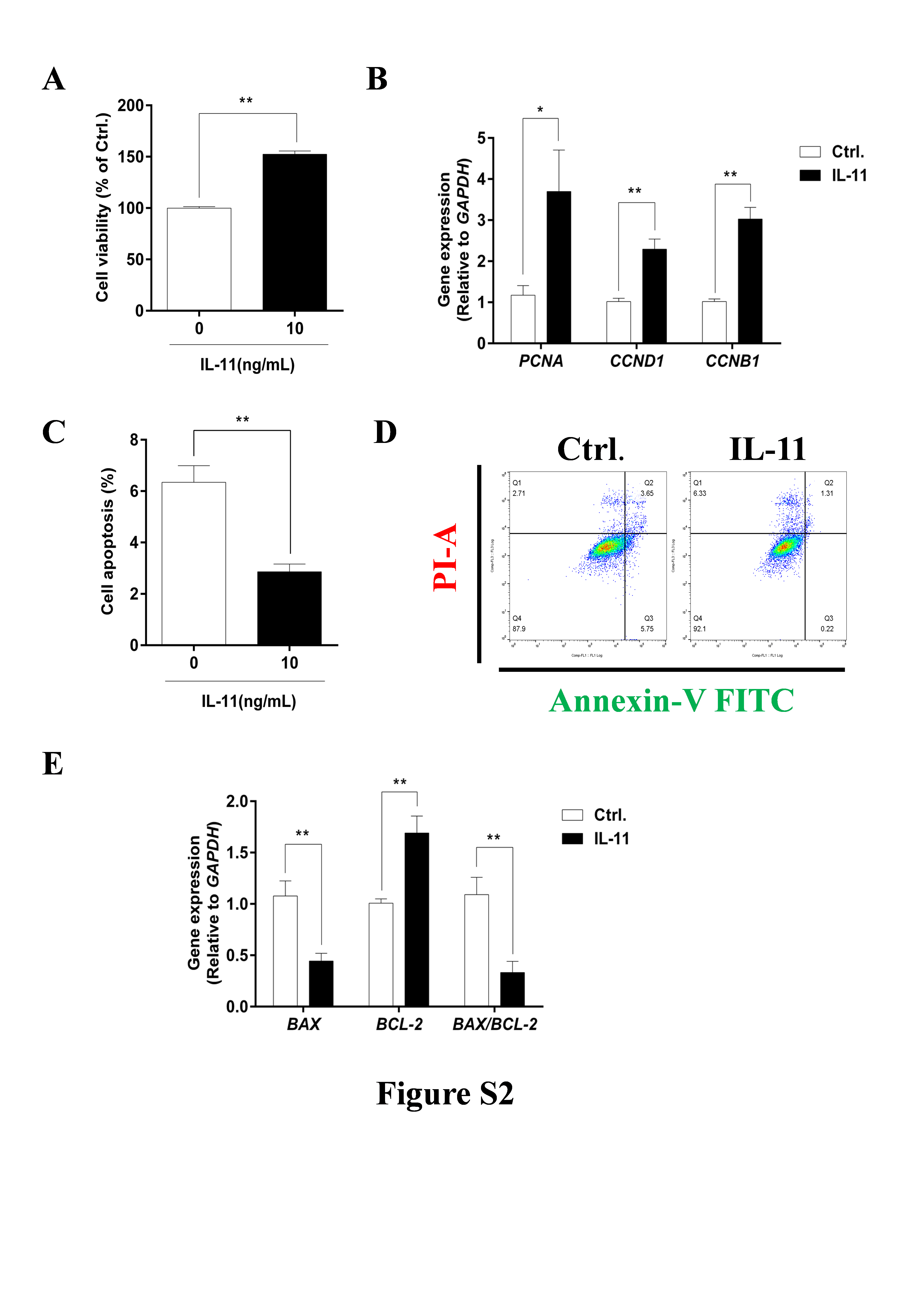

Supplement: Supplementary file 1 [file cells-12-00673-s001.zip › Figure S2.tif]
